# Molecularly engineered BODIPY photosensitizers for combined cancer phototherapy and immunotherapy

**DOI:** 10.3389/fphar.2026.1849977

**Published:** 2026-06-03

**Authors:** Huixia Wang, Yangming Zhang, Mengjiao Zhou

**Affiliations:** 1 The People’s Hospital of Danyang, Affiliated Danyang Hospital of Nantong University, Danyang, Jiangsu, China; 2 School of Pharmacy, Nantong University, Nantong, Jiangsu, China

**Keywords:** BODIPY photosensitizers, immunotherapy, photodynamic therapy, photothermal therapy, reactive oxygen species

## Abstract

BODIPY-based photosensitizers have attracted great interest in cancer phototherapy and immunotherapy due to their tunable structures, excellent photostability, and high molar extinction coefficients. However, conventional BODIPY dyes have inherent limitations, such as poor water solubility, shallow tissue penetration, oxygen dependence, and insufficient single-modality efficacy. To address these, recent studies have used molecular engineering (ring fusion, electronic modulation, supramolecular assembly, metal coordination) to enhance reactive oxygen species generation and photothermal conversion. Smart delivery systems with microenvironment responsiveness further enable tumor targeting and microenvironment remodeling. Importantly, BODIPY-mediated phototherapy combined with immunotherapeutic strategies (immune checkpoint blockade, pyroptosis/cuproptosis induction, cGAS-STING activation, etc.) achieves synergistic antitumor effects, transforming localized tumor ablation into systemic immunity. This review summarizes recent progress in molecularly engineered BODIPY photosensitizers, from molecular optimization and smart delivery to immune synergy, and discusses current challenges and future directions to promote their clinical translation.

## 1 Introduction

Cancer remains a leading cause of death worldwide, driving continuous research and optimization of various therapeutic strategies (Sonkin et al., 2024), including chemotherapy (Davodabadi et al., 2023; Yan M. et al., 2024), radiotherapy (Liao et al., 2024; Gong et al., 2025a; Verginadis et al., 2025), phototherapy (Zou et al., 2024a; Cai et al., 2025), and immunotherapy (Weng et al., 2022; Anders et al., 2023). Among these, phototherapy mainly consists of photodynamic therapy (PDT) and photothermal therapy (PTT) (Qi et al., 2024; Hou et al., 2020; Deng et al., 2021). In PDT, light activates a photosensitizer to generate reactive oxygen species (ROS), which effectively kill cancer cells (Zou et al., 2024b; Tao et al., 2025; Tao et al., 2026). In contrast, PTT uses photothermal agents to convert light into heat, raising the local temperature in the tumor (Li Y. et al., 2024; Nag et al., 2024; Wang Y. et al., 2024). This thermal effect causes denaturation and inactivation of biomolecules, ultimately leading to thermal ablation of cancer cells (Wei et al., 2024; Yan D. et al., 2024; Dai et al., 2025). Importantly, both PDT and PTT can trigger immunogenic cell death (ICD) (Wang S-Z. et al., 2023; Yu et al., 2023; Liu et al., 2024). During ICD, dying cancer cells release damage-associated molecular patterns (DAMPs), such as calreticulin (CRT), ATP, and high-mobility group box 1 (HMGB1) (Xu F. et al., 2024; Ma H. et al., 2025; Jiang et al., 2026). These signals recruit and activate dendritic cells, initiating a robust antitumor immune response that can lead to systemic tumor clearance (Rui et al., 2023; Zhang K. et al., 2023; Fang et al., 2025). The combination of phototherapy and photoimmunotherapy is increasingly crucial, as it addresses the limitations of single-modality therapies (Nag et al., 2024; Tavakkoli Yaraki et al., 2022). Single-modality phototherapy, though minimally invasive and locally precise, often fails to eradicate tumors completely and may induce recurrence/metastasis (Stoop et al., 2024; Zhang et al., 2025; Dai et al., 2026; Qi et al., 2025). Immunotherapy alone, while eliciting systemic immunity, has low response rates in solid tumors due to the immunosuppressive microenvironment (Galluzzi et al., 2023; Wang L. et al., 2024; Kong and Chen, 2022). Their synergy integrating local ablation with systemic immune activation enhances outcomes and induces long-term immune memory, representing a promising direction for advanced cancer treatment (Wu et al., 2023; Zhang Q. et al., 2024).

To fully harness these synergistic benefits, the development of high-performance organic photoactive agents with ideal photophysical and therapeutic properties has become a research priority (Overchuk et al., 2023; Ma D. et al., 2025; Han et al., 2023). In recent years, researchers have explored many types of organic photoactive agents for these therapies (Zhang Y. et al., 2024; Zhao et al., 2025; Gao et al., 2025). Common examples include boron-dipyrromethene (BODIPY) derivatives (Yu et al., 2022; Guan et al., 2025), porphyrins (Alhoussein et al., 2025; Anil Kuma et al., 2025; Liu et al., 2025), cyanine dyes (Kejík et al., 2024; Zhang J. et al., 2024; Yuan et al., 2025), and conjugated polymers, *etc* (Wang et al., 2020a; Wang et al., 2020b; Zheng et al., 2025). Notably, BODIPY-based compounds have attracted significant attention due to their excellent photophysical properties, strong therapeutic efficacy, and ability to induce ICD (Sun et al., 2024; Zhao et al., 2023). BODIPY is a class of structurally unique organic fluorescent dyes (Li W. et al., 2024; Chen et al., 2023). Their rigid, planar, conjugated framework endows them with a high molar extinction coefficient, excellent photostability, and versatile chemical tunability, making them highly promising candidates as photosensitizers for PDT and PTT (Ruan et al., 2023; Wang D. et al., 2023). However, the clinical translation of conventional BODIPY-based photosensitizers faces three inherent challenges. First, their strong hydrophobicity leads to poor bioavailability (Ge et al., 2024). Second, absorption in the visible range limits tissue penetration depth (Liu et al., 2020). Third, their reliance on oxygen (O_2_)-dependent Type II PDT mechanisms severely restricts efficacy in the hypoxic microenvironment of solid tumors (Paul et al., 2021). Together, these limitations act synergistically as major obstacles to the development of BODIPY-based photosensitizers, which may induce drug resistance and trigger compensatory survival pathways in cancer cells, thereby promoting tumor recurrence and metastasis.

To address these bottlenecks, researchers have pursued systematic advances along two complementary fronts: molecular engineering and nanoscale delivery (Du et al., 2025; Zhao et al., 2022). At the molecular level, cyclization imparts a rigid conformation that enables spontaneous self-assembly into stable nanoparticles, effectively overcoming aggregation-caused quenching (Wang J. et al., 2025). Introducing a formyl group fine-tunes electron distribution, enabling efficient dual Type I/II ROS generation without heavy atoms, even under hypoxia (Peng et al., 2024a). Meanwhile, coordination with cyclometalated Ir(III) leverages the heavy-atom effect to boost intersystem crossing, while also revealing how electron-transfer pathways dictate therapeutic outcomes (Paul et al., 2022; Peng et al., 2024b). On the delivery side, smart, stimuli-responsive carriers have endowed BODIPY with microenvironment-sensing capabilities (Zou et al., 2025; Zheng et al., 2026; Zou et al., 2026a). For example, fluorinated polymer backbones co-deliver O_2_ and photosensitizer to counteract tumor hypoxia (Zhang L. et al., 2024); hollow mesoporous organosilica nanoparticles consume glutathione to amplify oxidative stress (Yang et al., 2024); and hypoxia-responsive chitosan-based carriers enable fluorescence-guided drug release specifically in low O_2_ regions (Li et al., 2025). These synergistic innovations ensure precise tumor accumulation and maximized phototherapeutic effects.

As efficient tumor delivery and phototherapeutic performance are achieved, research focus has shifted to harnessing BODIPY-mediated phototherapy for systemic antitumor immunity, which synergizes with immunotherapy to amplify therapeutic outcomes (Liu et al., 2023). Immunotherapy has emerged as a prominent strategy in oncology in recent years (Gao et al., 2020; Hu et al., 2020; Guo J. et al., 2024), and its combination with phototherapy enables synergistic enhancement, significantly augmenting antitumor efficacy (Wu G. et al., 2024; Lu et al., 2024; Kah and Abrahamse, 2025). Early work demonstrated that combining PDT with immune checkpoint blockade is highly effective: PD-L1-targeted BODIPY nanoparticles not only eliminate primary tumors but also induce durable immune memory (Liu et al., 2021). New forms of immunogenic cell death have further expanded this paradigm (Wang J. et al., 2024). Cyclic BODIPY loaded with an IDO inhibitor triggers pyroptosis, while copper-coordinated BODIPY assemblies enhance cuproptosis, both significantly boosting immunogenicity and T-cell infiltration (Wen et al., 2025). Even more innovative are agonist-free strategies. Mitochondria-targeted nanoagonists induce mitochondrial DNA release upon irradiation, activating the cGAS-STING pathway without any exogenous agonist. Importantly, researchers have also uncovered a negative feedback loop: light irradiation can upregulate the COX-2/PGE2 pathway and PD-L1 expression (Zhang X. et al., 2023). By co-loading celecoxib, a COX-2 inhibitor, this immunosuppressive side effect can be turned into a therapeutic advantage. In summary, these advances have transformed BODIPY from a simple cytotoxic agent into a multifunctional platform for long-lasting antitumor immunity, integrating molecular optimization, smart delivery, and immune synergy to fulfill the core value of combined phototherapy and immunotherapy.

**FIGURE 1:**
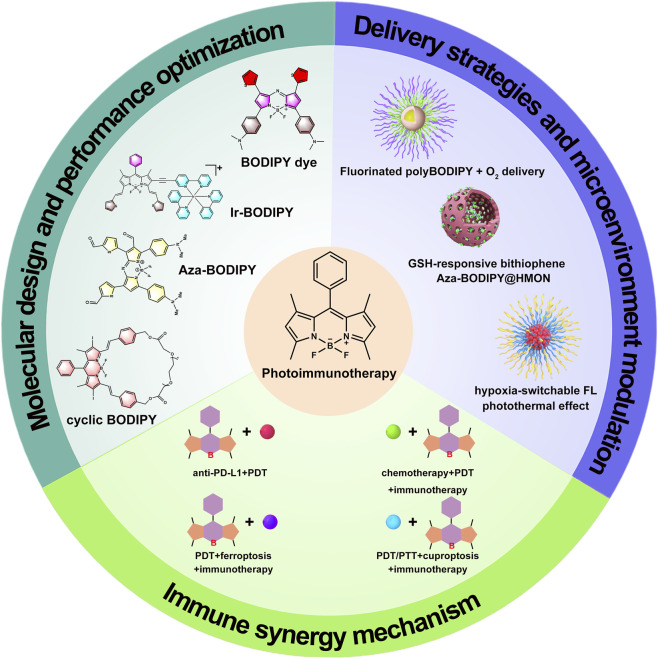
Schematic illustration of the molecularly engineered BODIPY-based nanoplatform with molecular design and performance optimization, delivery strategies and tumor microenvironment modulation, and immune synergy mechanism for antitumor therapy.

In recent years, BODIPY-based photosensitizers have advanced rapidly in cancer therapy, with the synergy of phototherapy and immunotherapy emerging as a core driving force. Different from most existing reviews that focus solely on single-modality phototherapy or isolated immune strategies, this review systematically integrates three key dimensions: molecular design optimization, intelligent delivery systems, and immune synergy mechanisms (Figure 1). It critically evaluates the advantages and limitations of various molecular engineering approaches, as well as the strengths and shortcomings of smart delivery systems and immune synergy strategies. Furthermore, this review offers a clear roadmap for the rational design of next-generation BODIPY-based theranostic platforms, facilitating their clinical translation and advancing the development of combined phototherapy-immunotherapy regimens.

## 2 Molecular engineering of BODIPY photosensitizers

**FIGURE 2:**
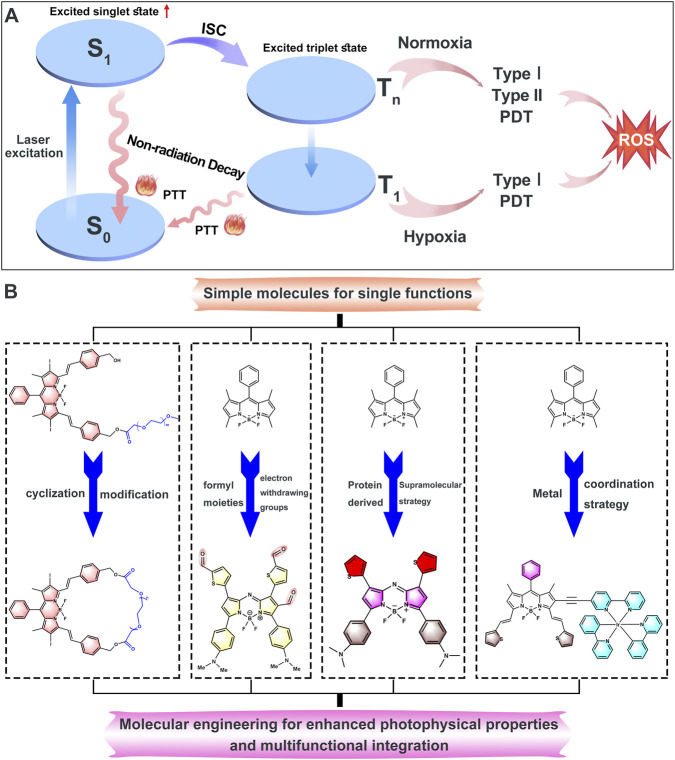
**(A)** Schematic illustration of the phototherapeutic mechanism of BODIPY photosensitizers. **(B)** Rational design of BODIPY molecules for multifunctional antitumor activity.

PDT has gained significant attention in cancer treatment due to its minimally invasive nature and spatiotemporal precision (Yang et al., 2023). At the heart of PDT efficacy lies the photosensitizer, and among various candidates, BODIPY dyes have emerged as a research hotspot owing to their tunable structure, excellent photostability, and high molar extinction coefficients (Lee et al., 2025). However, conventional BODIPY-based photosensitizers face several limitations that hinder clinical translation, such as strong hydrophobicity, shallow tissue penetration due to short absorption wavelengths, O_2_ dependency, and limited therapeutic outcomes from single-mode action. To overcome these challenges, recent studies have focused on molecular-level engineering through strategic chemical modifications. These approaches aim to modulate the excited-state behavior of BODIPY dyes, thereby enhancing ROS generation, red-shifting absorption wavelengths to improve tissue penetration, and endowing multifunctionality. Key strategies include cyclization (Li W. et al., 2023), electronic cloud modulation (Ma J. et al., 2025), supramolecular activation (Wang J. et al., 2023), and the heavy-atom effect (Li J. et al., 2023), all designed to address inherent drawbacks like poor solubility, low ROS yield, and insufficient near-infrared response. Notably, the phototheranostic performance of BODIPY-based photosensitizers is fundamentally a macroscopic manifestation of their excited-state behavior. From a photophysical perspective, after absorbing a photon, a photosensitizer transitions from its ground state (S_0_) to an excited singlet state (S_1_). From there, it can decay *via* several competing pathways: radiative relaxation (fluorescence), non-radiative relaxation (heat), or intersystem crossing (ISC) to the excited triplet state (T_1_). The triplet state then interacts with molecular O_2_ or cellular substrates through energy or electron transfer, generating ROS or heat (Figure 2A). Therefore, the central challenge in molecular design lies in deliberately steering these excited-state deactivation pathways, specifically, balancing the competition among fluorescence imaging, photothermal conversion, and photodynamic action to achieve optimal theranostic performance.

### 2.1 Molecular engineering strategies for overcoming BODIPY phototherapy limitations

This review highlights a clear evolutionary trajectory in BODIPY molecular engineering: from “passive adaptation” to “active control,” and from “single function” to “multifunctional integration.” Early efforts relied heavily on the heavy-atom effect to boost ISC. However, concerns over dark toxicity have limited its clinical potential. To address this limitation, researchers have developed a range of strategies for molecular engineering of BODIPY in recent years, including cyclization modification, formyl-based electronic cloud modulation, supramolecular activation, and Ir(III) metal coordination (Figure 2B).

Cyclization introduces conformational rigidity that suppresses intramolecular rotation, thereby minimizing non-radiative losses, and promotes ordered π-π stacking for enhanced photophysical performance (Wu J. et al., 2024). Notably, it exhibits adaptability to both hypoxic and normoxic environments due to its dual Type I/II ROS generation capability, producing singlet oxygen (^1^O_2_), superoxide anion (O_2_^−^), and hydroxyl radicals (OH), which enables effective PDT and immunogenic pyroptosis in cancer cells (Guo X. et al., 2024). Cyclization modification exhibits high ISC efficiency and low dark toxicity, achieves moderate tissue penetration *via* self-assembled nanoparticles, and is particularly suitable for tumors with heterogeneous oxygen levels (Zhang et al., 2026). Introducing electron-withdrawing groups like formyl moieties fine-tunes the electron density across the conjugated system, increasing the spin-orbit coupling constant and promoting ISC without any metal incorporation, thus avoiding heavy-atom-induced dark toxicity (Wang Z. et al., 2025). It also exhibits NIR absorption and generates both Type I/II ROS, making it a promising candidate for low-toxicity multifunctional phototherapy. Another elegant approach leverages supramolecular chemistry: BODIPY derivatives remain optically “silent” in their aggregated state but are selectively activated upon binding to serum albumin, a naturally abundant protein that acts as an endogenous “switch” (Jiang et al., 2025). This enables *in situ* control of excited-state behavior directly within the biological environment, improving tumor targeting and reducing off-target effects. This strategy generates Type II ROS and PTT effects after activation, and maintains very low dark toxicity in the aggregated state, thereby realizing spatiotemporally controlled PDT/PTT/immunotherapy in cancer cells. Metal coordination strategies still utilize the heavy-atom effect, but with greater precision (Long et al., 2022). By carefully selecting coordination sites and tuning intramolecular charge-transfer pathways, researchers have uncovered direct structure-property relationships between electronic structure and ISC efficiency, offering valuable guidance for rational design. While this strategy exhibits high therapeutic efficacy under NIR irradiation and enables subcellular targeting, it has moderate metal-induced dark toxicity.

Together, these molecular engineering approaches converge on a unified goal: precise control over how BODIPY dissipates its excited-state energy. The aim is to enable on-demand allocation of output, whether fluorescence for imaging, heat for photothermal therapy, or ROS for photodynamic action under irradiation. Such tailored photophysics lays the essential molecular foundation for subsequent advances in smart delivery and immune-synergistic therapy. When directly comparing these strategies, formyl modification stands out for its heavy-atom-free feature and very low dark toxicity, while cyclization modification is superior in adapting to the heterogeneous oxygen environments of tumors. In contrast, supramolecular activation possesses unique advantages in spatiotemporal control via endogenous albumin, whereas metal coordination excels in subcellular targeting and high therapeutic efficacy despite its moderate metal-induced dark toxicity.

### 2.2 Molecularly engineered BODIPY in combined phototherapy and photoimmunotherapy

To better highlight how rational molecular design of BODIPY dyes enables multifunctionality, and to emphasize their combined advantages in phototherapy and immunotherapy for cancer treatment, Table 1 summarizes some representative studies. It outlines the design strategies, key physicochemical properties, and antitumor efficacy of various BODIPY-based photosensitizers. These examples not only illustrate how structural modifications influence phototherapeutic performance but also reveal the structure-function relationships critical for developing integrated theranostic agents for combined phototherapy and photoimmunotherapy.

**TABLE 1:** Representative BODIPY-based nanoplatforms for combined phototherapy and immunotherapy in cancer treatment.

Nanomaterial	Therapeutic method	Design advantages	Property and therapeutic effect	Ref
BRGNs	PDT, immunogenic pyroptosis	cyclization modification	size = 108 nm, negative ζ-potentials, various ROS (^1^O_2_, O_2_^−^, OH), 4T1 cells	Wen et al. (2025)
BDP-6@F127	PDT/PTT, ICB	formylated modification	size = 106.8 nm, 808 nm, type-I and type-II PDT, Cal-33 cells	Peng et al. (2024a)
B4-BSA	PDT/PTT, immunotherapy	protein-derived supramolecular strategy	B4 NPs (size ≈ 220 nm), B4-BSA (size ≈ 164 nm), 808 nm, 4T1 cells	Cheng et al. (2022)
Ir-1/Ir-2	PDT, immunotherapy	cyclometalated modification	630 nm, 0.6 W cm^-2^, A549 cells	Xu N. et al. (2024)
NP@Poly^FBODIPY^	PDT, immunotherapy	delivering O_2_, hypoxia relief	size ≈ 90 nm, 650 nm, 5 min, 0.1 W/cm^2^, 4T1 cells	Zhang L. et al. (2024)
Aza-BODIPY@HMON	PDT/PTT, immunotherapy	GSH-responsive	size ≈ 150–200 nm, 808 nm, 4T1 cells	Yang et al. (2024)
CsBPNs	PTT, immunotherapy	hypoxia-responsive, hypoxia imaging	size ≈ 100.7 ± 6.3 nm, zeta = 21.0 ± 1.6 mV, 680 nm, 0.5 W/cm^2^, 30 s, 4T1 cells	Li et al. (2025)
BODIPY-Caged Resiquimod	PDT, immunotherapy	photocaging site for resiquimod	660 nm LED,HeLa cells and A549 cells	Roshdy et al. (2025)
mt-NP^Bodipy^	PDT, immunotherapy	cancer imaging, ROS-responsive	size = 92 nm, zeta = −11 mV, 808 nm, CT26 cells	Tang et al. (2024)
BDP-I-N-anti-PD-L1	PDT, immunotherapy	luminescence in the NIR-II window (1000–1700 nm)	size ≈ 10.1 nm, 740 nm, 150 mW/cm^2^, MC38 cells	Liu et al. (2021)
NP2, BODIPY, platinum complex	PDT, immunotherapy, chemotherapy	NIR light-responsive release	size ≈ 86.8 nm, 808 nm, 1 W/cm^2^, 4T1 cells	Wang B. et al. (2023)
HABH	PDT, immunotherapy	agonist independent cGAS-STING activation	type I PDT, size ≈ 150 nm, POD-like activity, 4T1 cells	Xu et al. (2025)
BCuNP@NM	PDT/PTT, cuproptosis, immunotherapy	NK cell membrane camouflaged	size ≈ 210.6 ± 4.1 nm, zeta = −14.8 ± 1.7 mV, 658 nm (PDT), 1064 nm (PTT), 4T1 cells	Yu et al. (2025)
NP^PDT^@CXB	PDT, immunotherapy	ROS-responsive	size ≈ 115.2 nm, 808 nm, ovarian cancer	Zhang X. et al. (2023)

**FIGURE 3:**
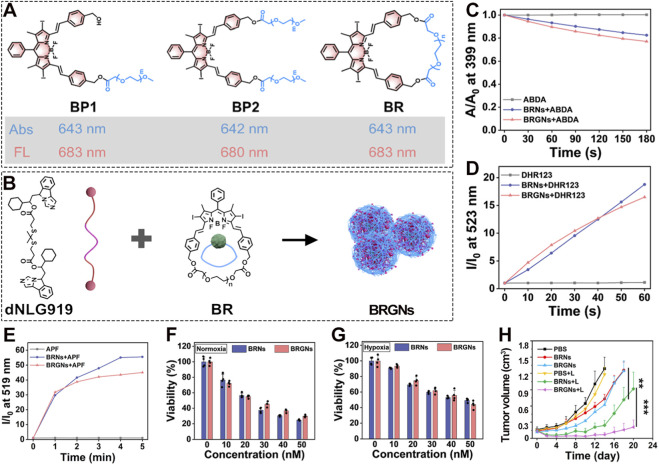
**(A)** Structures of BP1, BP2, and BP3 photosensitizers and their UV-vis absorption and fluorescence emission data. **(B)** Preparation step of BRGNs. **(C–E)** Detection of ROS using specific probes: **(C)** ABDA for ^1^O_2_, **(D)** DHR123 for superoxide anion, and **(E)** APF/DHR123 for OH. **(F,G)** *In vitro* cytotoxicity under **(F)** normoxic and **(G)** hypoxic conditions. **(H)** Tumor volume changes in tumor-bearing mice. Reproduced with permission from Wen et al., 2025. Copyright 2025 American Chemical Society.

For instance, Wen and colleagues developed a cyclic BODIPY photosensitizer (denoted BR) (Wen et al., 2025). The rigid conformation imparted by the ring structure enabled BR molecules to spontaneously self-assemble into stable nanoparticles *via* π-π stacking and hydrogen bonding (Figure 3A). This cyclization strategy effectively prevented aggregation-caused quenching, a common issue with linear analogs, and significantly improved bioavailability. Moreover, the linear and cyclic molecules exhibit identical UV absorption and emission. Under light irradiation, BR efficiently generated ROS, laying the groundwork for subsequent formulation into BRGNs by loading an IDO inhibitor to induce pyroptosis (Figure 3B). Using specific ROS scavengers, the authors confirmed that BRGNs produced multiple ROS species, including ^1^O_2_ (Figure 3C), O_2_^−^ (Figure 3D), and OH (Figure 3E). By combining both Type I (electron transfer) and Type II (energy transfer) mechanisms, BRGNs maintained anticancer activity under both normoxic (Figure 3F) and hypoxic conditions (Figure 3G). *In vivo* tumor suppression experiments further demonstrated that mice treated with BRGNs plus light exhibited the smallest tumor volumes compared to all control groups (Figure 3H), fully verifying the synergistic effect between cyclization-modified BODIPY-mediated PDT and immunogenic pyroptosis. This work underscores how rational topological design can fundamentally enhance the physicochemical properties of BODIPY dyes.

**FIGURE 4:**
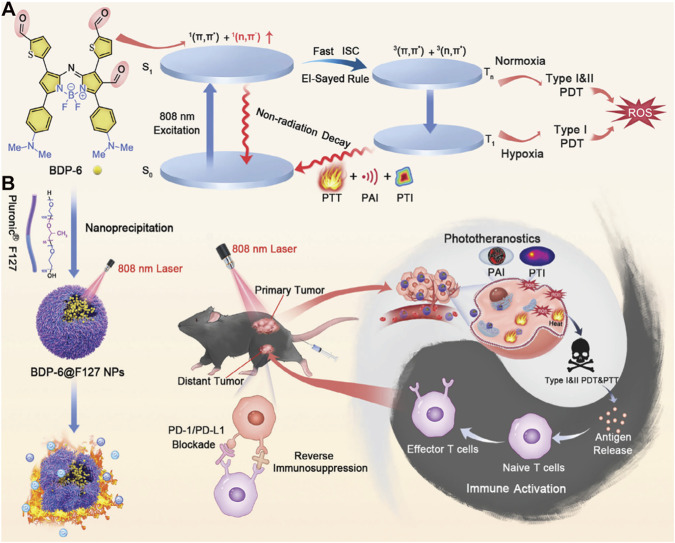
**(A)** Photophysical and photochemical mechanism of BDP-6 photosensitizer. **(B)** Preparation of BDP-6@F127 NPs and their combined antitumor effect. Reproduced with permission from Peng et al., 2024a.

While cyclization improves stability, boosting ROS generation without introducing heavy atoms remains a separate challenge. Addressing this, Peng *et al.* designed a formyl-modified Aza-BODIPY derivative that enables dual Type I/II PDT without relying on heavy atoms (Peng et al., 2024a). By incorporating a formyl group onto the Aza-BODIPY core, they precisely tuned the conjugation length and electron distribution (Figure 4A). This modification enhanced spin-orbit coupling, promoting efficient intersystem crossing even in the absence of heavy atoms. The resulting dye, BDP-6, exhibited robust capabilities in generating both Type I (*e.g.*, OH under hypoxia) and Type II ROS, along with a remarkable photothermal conversion efficiency of 50.13%. Moreover, BDP-6 self-assembled with F127 polymer to form BDP-6@F127 NPs (Figure 4B). This nanoplatform demonstrated efficient combined photodynamic, photothermal, and immunotherapeutic effects, effectively inhibiting Cal-33 cancer cell proliferation. This formylation modification strategy breaks away from the traditional “heavy-atom paradigm” and provides a promising route toward low dark-toxicity, multifunctional photosensitizers for combined photoimmunotherapy.

**FIGURE 5:**
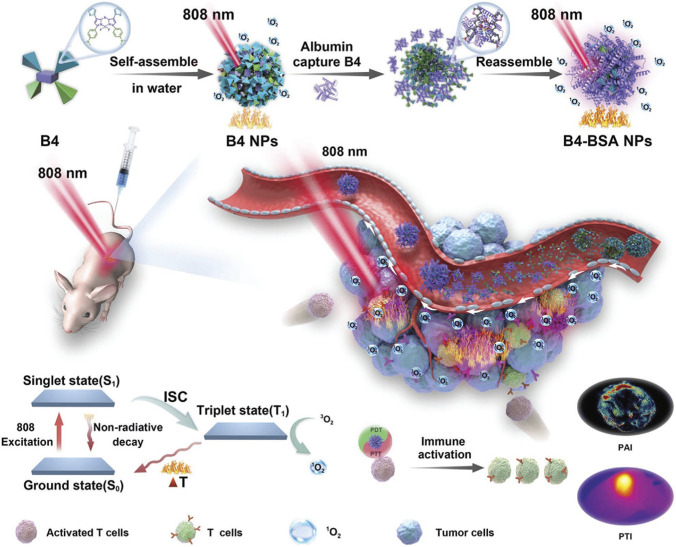
Preparation of B4-BSA NPs and their mechanism for inhibiting cancer cell proliferation under 808 nm laser irradiation. Reproduced with permission from Cheng et al., 2022. Copyright 2022 Wiley-VCH GmbH.

Beyond covalent modifications, researchers have also leveraged supramolecular host-guest interactions to achieve “on-demand” activation of BODIPY performance. For example, Cheng et al. constructed heavy-atom-free BODIPY-based nanoparticles (B4 NPs) that remain optically silent in their aggregated state, namely, their fluorescence and photothermal activity fully suppressed (Cheng et al., 2022). Upon intravenous injection, B4 NPs disassemble *in vivo*, releasing monomeric BODIPY molecules that selectively bind to serum albumin (Figure 5). This binding event simultaneously restores fluorescence and activates photothermal conversion. By exploiting endogenous albumin as a biological “switch,” this strategy not only simplifies synthesis but also achieves spatiotemporally controlled theranostics, laying a foundation for precise combined phototherapy and immunotherapy and showcasing the unique power of supramolecular chemistry in photosensitizer design.

**FIGURE 6:**
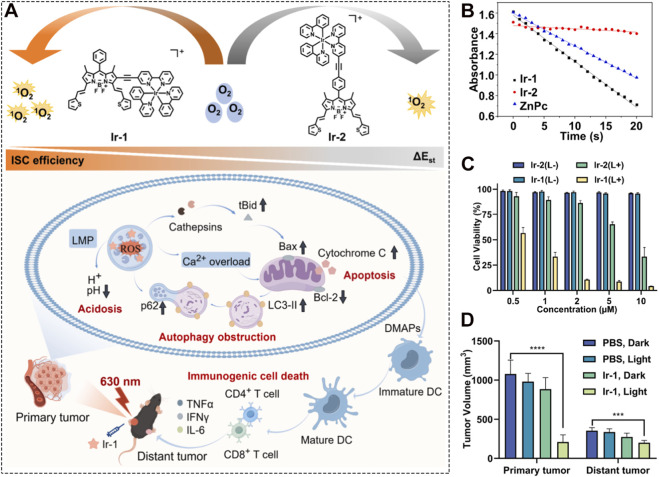
**(A)** Molecular structures of Ir-1 and Ir-2 and schematic illustration of their combined phototherapy and immunotherapy for inhibiting cancer cell proliferation. **(B)** Comparison of ROS generation capability among Ir-1, Ir-2, and ZnPc. **(C)** Cytotoxicity of Ir-1 and Ir-2 with and without light irradiation. **(D)** Tumor volume changes in mice after treatment with Ir-1 or Ir-2. Reproduced with permission from Xu N. et al., 2024. Copyright 2024 Elsevier B.V.

In addition to purely organic modifications, metal coordination offers another powerful avenue. Incorporating metal complexes, particularly through the heavy-atom effect, can further fine-tune excited-state dynamics and enable subcellular targeting for enhanced antitumor effects (Zhang D. et al., 2023). For example, Su *et al.* synthesized two isomeric BODIPY-Ir(III) conjugates (Ir-1 and Ir-2) differing only in the attachment site of the cyclometalated Ir(III) fragment (Xu N. et al., 2024). They found that when Ir(III) was directly linked to the BODIPY core via a conjugated bond (Ir-1), electron transfer was maximized, leading to dramatically accelerated intersystem crossing and superior ROS generation compared to the isomer with Ir(III) attached at the meso-phenyl position (Ir-2). Mechanistic studies revealed that after cellular uptake, Ir-1 specifically accumulated in lysosomes (Figure 6A). Upon irradiation, it induced lysosomal membrane permeabilization, disrupted calcium homeostasis, and triggered lysosome-dependent cell death. Using the DPBF probe, the authors compared ^1^O_2_ production among Ir-1, Ir-2, and ZnPc (a standard photosensitizer); Ir-1 caused the most pronounced DPBF degradation, confirming its exceptional ^1^O_2_ yield (Figure 6B). Consistently, cytotoxicity assays showed that Ir-1 achieved significantly lower cell viability under identical conditions (Figure 6C). Given its strong phototherapeutic effect and PDT’s ability to stimulate immune responses, the team further evaluated its impact on both primary and distant tumors (Figure 6D). Results confirmed that Ir-1 plus light treatment led to marked suppression of both tumor types, demonstrating effective photo-immunotherapy. This study not only develops a high-performance near-infrared photosensitizer but also clarifies the structure-function relationship between metal coordination sites and therapeutic efficacy, providing guidance for designing BODIPY-based combined photoimmunotherapy systems.

## 3 Smart delivery and microenvironment modulation for BODIPY nanoplatform

**FIGURE 7:**
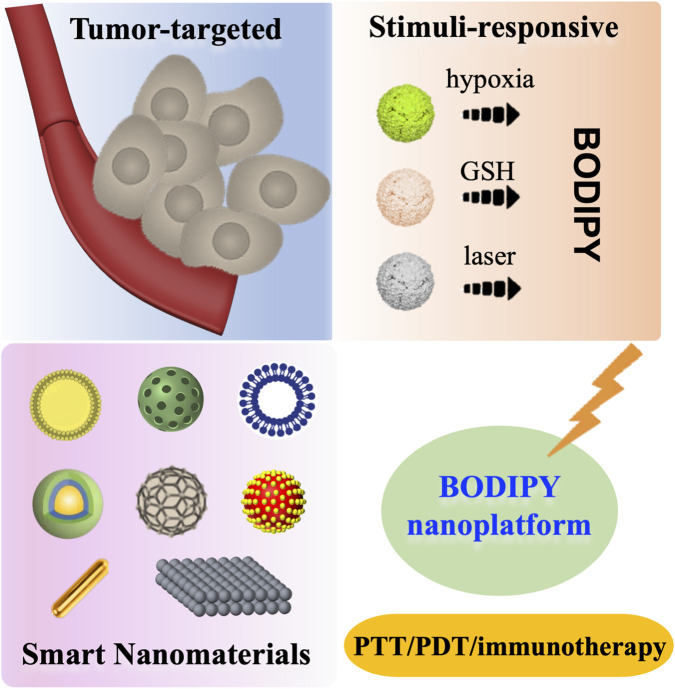
Schematic illustration of BODIPY nanoplatforms for smart delivery and tumor microenvironment modulation.

PDT for cancer is a complex process. It depends not only on the molecular structure of the photosensitizer but also on the intricate tumor microenvironment (Shi et al., 2023; Wang Y. et al., 2023; Cui et al., 2025). While optimizing the molecular properties of BODIPY dyes provides a solid foundation for effective phototherapy, their *in vivo* delivery still faces major hurdles, poor water solubility, insufficient tumor targeting, and the heterogeneous nature of the tumor microenvironment (He and Ma, 2024; Niu et al., 2021; Yang et al., 2021). Molecularly engineered BODIPY photosensitizers are BODIPY derivatives subjected to molecular-level modification, such as functional group substitution, with the primary aim of optimizing their photophysical and photochemical properties. In contrast, BODIPY nanoplatforms function as delivery systems that integrate molecularly engineered BODIPY (serving as the photosensitizer core) with carrier materials, including polymers and mesoporous silica, to realize smart delivery and tumor microenvironment modulation (Figure 7). Recent studies have demonstrated that drug delivery systems engineered to target the tumor microenvironment play a pivotal role in modulating the therapeutic efficacy of drug molecules against a wide range of diseases (Jie et al., 2021; Shen et al., 2022; Xia et al., 2023). Therefore, developing intelligent delivery systems that enable specific accumulation and controlled release of BODIPY at tumor sites, while simultaneously modulating and exploiting the tumor microenvironment, has become the next critical step toward clinical translation.

**FIGURE 8:**
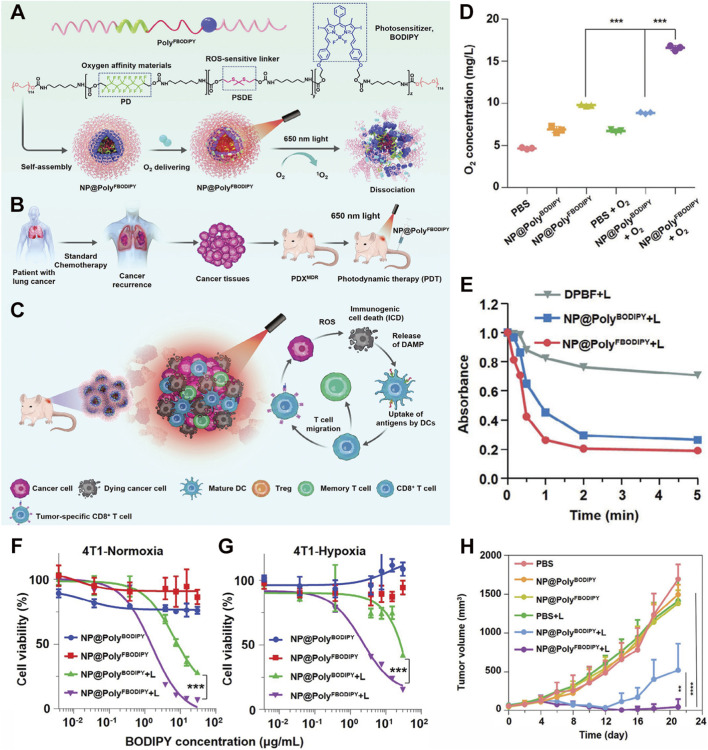
**(A)** Preparation steps of the NP@PolyFBODIPY nanoplatform. **(B)** Schematic illustration of PDT treatment of recurrent tumors using the NP@PolyFBODIPY nanoplatform. **(C)** Schematic illustration of the immunotherapeutic mechanism of the NP@PolyFBODIPY nanoplatform. **(D)** Comparison of O_2_ storage capacity among different experimental groups. **(E)** Comparison of ROS generation capability. **(F,G)** Cytotoxicity of the nanoplatform against 4T1 cancer cells under **(F)** normoxic and **(G)** hypoxic conditions. **(H)** Tumor volume changes in mice across different experimental groups. Reproduced with permission from Zhang L. et al., 2024. Copyright 2024 Wiley-VCH GmbH.

Hypoxic microenvironments are a major obstacle to phototherapy efficacy (Yi et al., 2023; Hao et al., 2024; Li et al., 2026; Cheng et al., 2021). To tackle hypoxia, a hallmark of solid tumors, Zhang *et al.* co-polymerized a BODIPY photosensitizer with O_2_-affinic fluorinated alkyl chains to form self-assembling PolyFBODIPY nanoparticles (Figure 8A) (Zhang L. et al., 2024). This BODIPY nanoplatform simultaneously delivers both the photosensitizer and O_2_. Upon irradiation at 650 nm, it generates ROS while rapidly disassembling *via* cleavage of ketal bonds, thereby releasing stored O_2_ to alleviate tumor hypoxia (Figure 8B). Mechanistic studies revealed that this approach reprograms lipid and amino acid metabolism in cancer cells, restoring sensitivity to PDT in multidrug-resistant tumors (Figure 8C). As a result, PolyFBODIPY nanoparticles achieved potent combined photodynamic and immunotherapeutic effects against 4T1 tumor. To confirm the role of the fluorocarbon segment in O_2_ delivery, the authors compared the O_2_-carrying capacity of NP@PolyFBODIPY with that of NP@PolyBODIPY (lacking fluorination) under standard or O_2_-enriched conditions. The results clearly showed that NP@PolyFBODIPY stored significantly more O_2_ (Figure 8D). Furthermore, using the ROS probe DPBF, they demonstrated that NP@PolyFBODIPY produced more ^1^O_2_ than its non-fluorinated counterpart (Figure 8E). Cytotoxicity assays under normoxic (21% O_2_) and hypoxic (1% O_2_) conditions confirmed that NP@PolyFBODIPY was far more effective at killing 4T1 cells, exhibiting a much lower IC_50_ value (Figures 8F,G). *In vivo*, a single treatment with NP@PolyFBODIPY nearly eradicated 4T1 tumors in mice, outperforming all control groups (Figure 8H). This work highlights the great potential of carrier engineering in BODIPY nanoplatform to overcome hypoxia, the inherent bottleneck of PDT.

**FIGURE 9:**
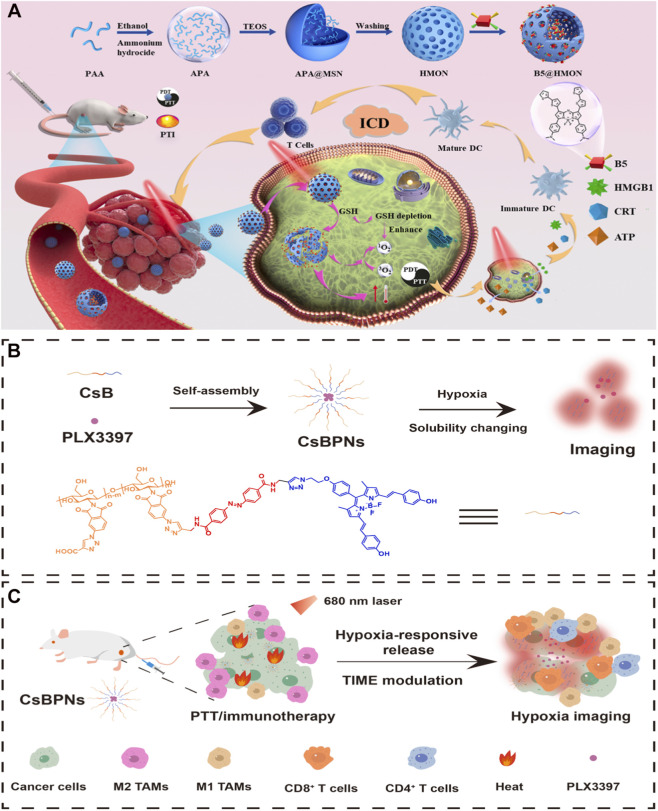
**(A)** Preparation of B5@HMON nanoplatform and its schematic illustration for antitumor therapy. Reproduced with permission from Ref. (Yang et al., 2024). Copyright 2024 Elsevier B.V. **(B)** Molecular structure of CsB and preparation steps of the CsBPNs nanoplatform. **(C)** Schematic illustration of the phototherapy and immunotherapy mediated by the CsBPNs nanoplatform under 680 nm irradiation for antitumor treatment. Reproduced with permission from Li et al., 2025. Copyright 2024 Elsevier Inc.

Hypoxia and high levels of intracellular reducing agents like glutathione (GSH) are two key factors that limit PDT efficacy (Gong et al., 2025b; Wang H. et al., 2023; Dangi et al., 2025). While co-delivering O_2_ can mitigate hypoxia, actively depleting reducing substances offers another powerful strategy (Xiong et al., 2021). Based on this, Wang *et al.* addressed this by loading a molecularly engineered bithiophene-aza-BODIPY (B5) into hollow mesoporous organosilica nanoparticles (HMONs) containing disulfide bonds (Figure 9A) (Yang et al., 2024). The porous structure of HMONs not only improved B5’s water solubility and drug-loading capacity but also enhanced light harvesting and O_2_ adsorption, boosting PDT efficiency. More importantly, in the GSH-rich tumor microenvironment, HMONs degraded and consumed GSH while releasing B5, amplifying oxidative stress without any chemotherapeutic agent. This BODIPY nanoplatform enabled synergistic PDT, PTT, and immunotherapy, leading to complete tumor eradication in the 4T1 model. This study demonstrates that the carrier in BODIPY nanoplatform is not just a passive delivery vehicle, it can actively reshape the tumor microenvironment to enhance therapeutic outcomes.

Beyond directly alleviating hypoxia or scavenging GSH, researchers have also exploited the unique features of the tumor microenvironment to design stimuli-responsive BODIPY nanoplatforms (Cheng et al., 2024; Jia et al., 2026; Sun et al., 2025). For example, Gao *et al.* conjugated a molecularly engineered BODIPY derivative to chitosan *via* an azobenzene-based hypoxia-cleavable linker and co-loaded the immunomodulator PLX3397, forming the CsBPNs nanoplatform (Figure 9B) (Li et al., 2025). Under normoxic conditions, the nanoparticles remained “silent.” But once they reached hypoxic regions, the azo bond broke, restoring fluorescence and triggering drug release (Figure 9C). This design enabled real-time imaging of hypoxic zones while precisely delivering PLX3397 to tumor-associated macrophages (TAMs) in those areas. As a result, M2-like immunosuppressive TAMs were repolarized toward the pro-inflammatory M1 phenotype, reversing immune suppression. This work exemplifies the “theranostic” concept by integrating imaging-guided therapy with therapy-validated imaging.

**FIGURE 10:**
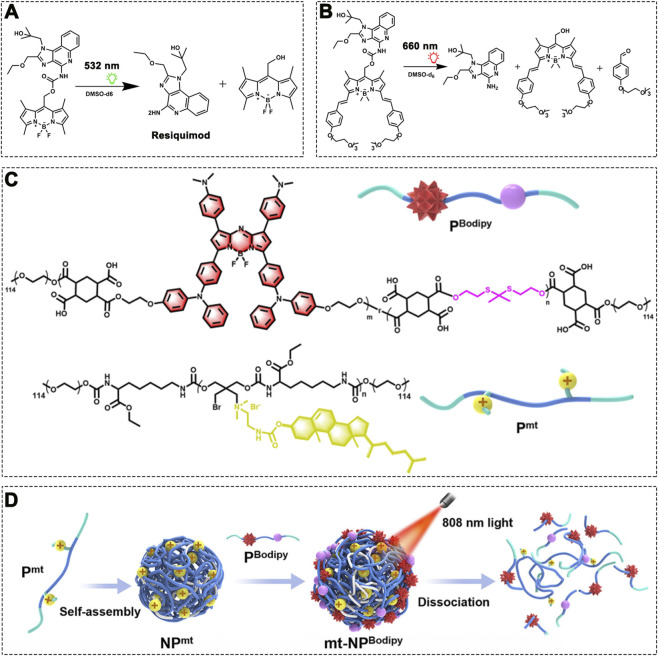
**(A)** Molecular structure of the 532 nm-absorbing and **(B)** 660 nm-absorbing BODIPY-resiquimod conjugates and schematic illustration of resiquimod release upon light irradiation. Reproduced with permission from Ref. (Roshdy et al., 2025). Copyright 2024 Springer Nature. **(C)** Molecular structure of the BODIPY-based polymer. **(D)** Self-assembly process of the BODIPY polymer into the mt-NPBodipy nanoplatform. Reproduced with permission from Tang et al., 2024.

In addition to endogenous triggers like hypoxia, external stimuli can serve as remote controls for spatiotemporally precise drug activation, taking light-controlled therapy to a new level (Zou et al., 2026b). External triggers include light, ultrasound, and X-rays. Abe *et al.* covalently linked the TLR7/8 agonist resiquimod to a BODIPY photosensitizer, creating two “caged” prodrugs (Roshdy et al., 2025). Due to differences in BODIPY conjugation, one prodrug responded to green light (Figure 10A), the other to red light (Figure 10B). Molecular docking helped identify the optimal caging site, ensuring the agonist remained completely inactive in the dark. Upon red-light irradiation, BODIPY not only generated ROS for PDT but also released resiquimod to activate immune responses. This dual-function design solved two problems at once: it prevented off-target toxicity of the immune agonist and masked the dark toxicity of BODIPY. Critically, both therapeutic actions, PDT and immune activation, were integrated into a single molecule, achieving precise spatiotemporal coordination.

While such molecular-level photocontrol can mitigate drug-related toxicity, it’s important to remember that nanoplatforms must first reach and be internalized by cancer cells to work as intended. Thus, how these carriers approach and cross the cell membrane is crucial. Researchers have found that cationic polymers can synergize with PDT to enable membrane-targeted photoimmunotherapy. For example, Xiao *et al.* designed a cationic polymer (Pmt) that targets the cell membrane and combined it with a degradable BODIPY-containing polymer (PBodipy) to form mt-NPBodipy nanoparticles (Figure 10C) (Tang et al., 2024). During circulation, the nanoparticle surface charge was shielded to reduce nonspecific interactions. Once at the tumor site, electrostatic attraction allowed the particles to anchor onto and disrupt the cancer cell membrane. Upon light irradiation, BODIPY-generated ROS further intensified lipid peroxidation and membrane rupture (Figure 10D). This dual-membrane-damage mechanism not only killed tumor cells efficiently but also released abundant damage-associated molecular patterns (DAMPs), which acted as endogenous adjuvants to activate dendritic cells. This study reveals that cationic polymers themselves can function as immune adjuvants, working in concert with BODIPY-based PDT to significantly amplify photoimmunotherapeutic efficacy.

## 4 Immune synergy and therapeutic innovation

**FIGURE 11:**
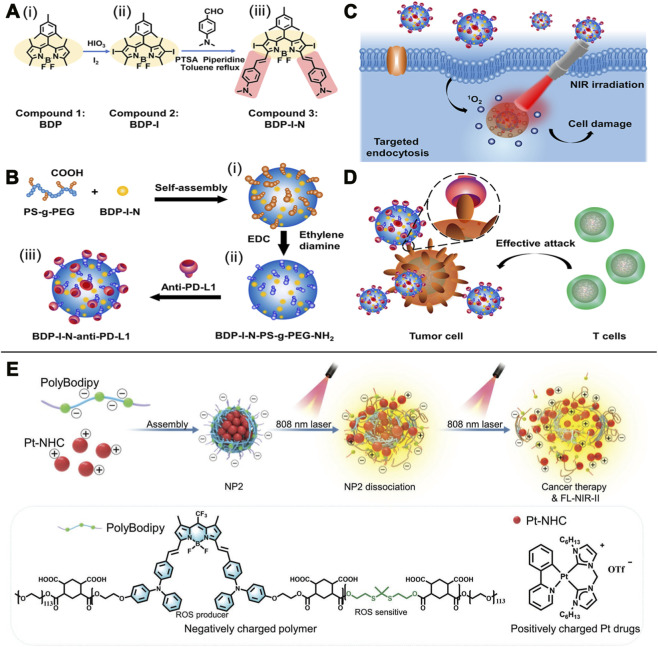
**(A)** Synthetic route of BDP-I-N molecule. **(B)** Design strategy for the BDP-I-N–anti-PD-L1 nanoplatform. **(C)** Schematic illustration of phototherapeutic efficacy of the nanoplatform under light irradiation to suppress cancer cell viability. **(D)** Immunotherapeutic efficacy of the nanoplatform. Reproduced with permission from Ref. (Liu et al., 2021). Copyright 2021 American Chemical Society. **(E)** Molecular structures of PolyBodipy and Pt-NHC, and schematic illustration of their assembly into NPs and dissociation to release the chemotherapeutic drug under 808 nm irradiation. Reproduced with permission from Wang B. et al., 2023. Copyright 2023 Wiley-VCH GmbH.

To achieve effective tumor treatment, the integration of BODIPY-based phototherapy with immune regulation has become a key research direction in oncology. Once BODIPY-based photosensitizers can precisely reach tumors and efficiently generate ROS, researchers have uncovered an even more powerful effect: PDT not only kills cancer cells directly but also triggers ICD, thereby activating the body’s antitumor immune response. This insight has given rise to a new paradigm, photoimmunotherapy. A key frontier in current research is how to deeply integrate BODIPY-mediated phototherapy with immune-modulating strategies for synergistic outcomes. To this end, Jiang *et al.* developed a heavy-atom-containing BODIPY photosensitizer, BDP-I-N (Figure 11A) (Liu et al., 2021). This compound was then conjugated with a PD-L1 monoclonal antibody onto the surface of BODIPY nanoparticles, creating an integrated theranostic nanoplatform named BDP-I-anti-PD-L1 NPs (Figure 11B). The authors demonstrated that these nanoparticles were efficiently internalized by cancer cells (Figure 11C). Under 808 nm irradiation, PDT eradicated the primary tumor, while the anti-PD-L1 antibody simultaneously blocked the PD-1/PD-L1 checkpoint, reactivating adaptive immunity (Figure 11D). Notably, this probe also emitted strong fluorescence in the NIR-II window, enabling real-time imaging of PD-L1 expression *in vivo*, with a tumor-to-normal tissue ratio as high as 14.1. This work shifts BODIPY-based therapy from simple tumor ablation to the induction of durable immune memory, laying a foundation for synergistic tumor treatment. BODIPY-mediated PDT induces ICD to release tumor antigens, and the anti-PD-L1 antibody reactivates T cells, forming a synergistic cycle that enhances antitumor immunity and sustains therapeutic effects.

Beyond antibody-mediated immune activation, ROS generated during PDT itself can serve as potent ICD inducers, naturally bridging phototherapy and immunotherapy. Chemotherapy can also trigger ICD, offering another route to immune engagement. Building on this, Xiao *et al.* designed an amphiphilic PolyBodipy polymer that self-assembled into nanoparticles and electrostatically encapsulated a cationic Pt-NHC complex (Figure 11E) (Wang B. et al., 2023). This system combined both a photosensitizer and a chemotherapeutic agent in one platform. Mechanistic studies revealed a dual ICD strategy: under 808 nm light, PolyBodipy produced ROS to induce Type II ICD; in the absence of light, the nanoparticles gradually disassembled, releasing Pt-NHC, which independently acted as a Type II ICD inducer. Thanks to this complementary, light-dependent and light-independent ICD mechanism, the system nearly eliminated triple-negative breast tumors even at low doses. Importantly, this design illustrates that true synergy between chemo- and phototherapy should go beyond simple functional addition; it must achieve biological reinforcement through complementary mechanisms. Such biological reinforcement is achieved through complementary ICD induction, where BODIPY-mediated PDT triggers light-dependent ICD and Pt-NHC release induces light-independent ICD to jointly enhance immune activation and tumor killing efficiency.

**FIGURE 12:**
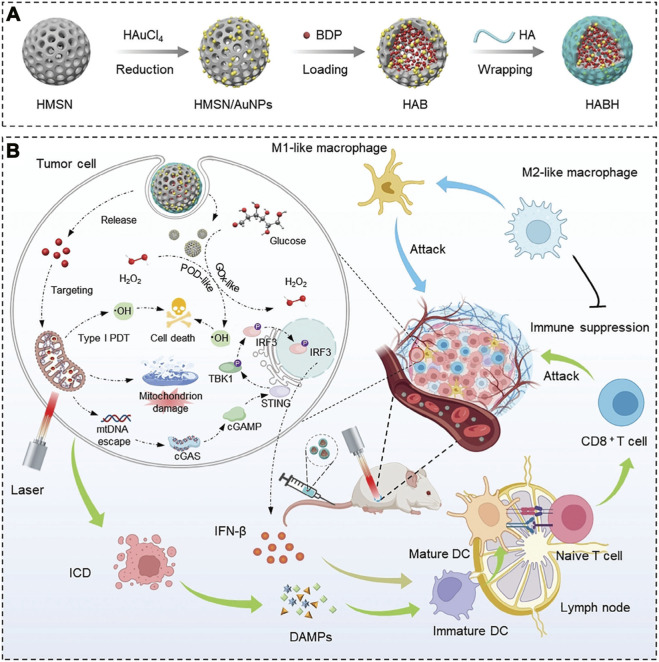
**(A)** Preparation of HABH nanoplatform. **(B)** Schematic illustration of the combined phototherapy and immunotherapy mediated by the HABH nanoplatform for antitumor treatment. Reproduced with permission from Xu et al., 2025. Copyright 2025 Wiley-VCH GmbH.

BODIPY is not limited to Type II PDT; it can also drive robust Type I PDT, which activates innate immune pathways such as cGAS-STING, without requiring external agonists. For example, Xu *et al.* exemplified this by first fabricating hollow mesoporous silica nanoparticles decorated with gold nanoparticles (HMSN/AuNPs), then loading them with a mitochondria-targeting BODIPY to form the HABH nanoagonist (Figure 12A) (Xu et al., 2025). Upon irradiation, this BODIPY generated OH inside mitochondria, causing mitochondrial stress and releasing mitochondrial DNA (mtDNA) into the cytosol (Figure 12B). Meanwhile, the HMSN/AuNPs exhibited nanozyme-like activity that amplified oxidative damage in a cascade manner. Together, these effects robustly activated the cGAS-STING pathway and promoted type I interferon production, without any exogenous STING agonist. This agonist-free design leverages mitochondrial mtDNA release and nanozyme-enhanced oxidative stress to activate innate immunity, avoiding the systemic toxicity associated with synthetic STING agonists.

**FIGURE 13:**
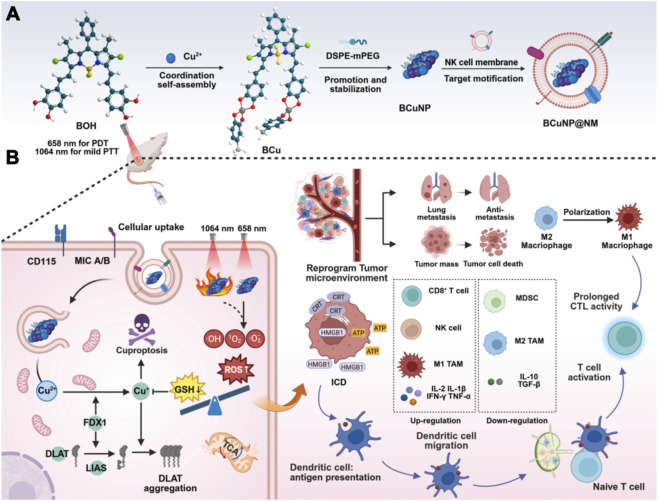
**(A)** Preparation of the BCuNP@NM nanoplatform. **(B)** Schematic illustration of the combined PDT, PTT, and immunotherapy mediated by the BCuNP@NM nanoplatform under 658 nm and 1064 nm irradiation for antitumor treatment. Reproduced with permission from Yu et al., 2025. Copyright 2025 Elsevier B.V.

In addition to PDT-induced ICD, cuproptosis, a recently identified form of copper-dependent programmed cell death, has drawn attention for its ability to trigger ICD and stimulate antitumor immunity. Combining PDT with cuproptosis offers not only direct tumor killing but also enhanced ICD for stronger antiproliferative effects. For example, Xiong *et al.* capitalized on this by coordinating catechol-functionalized BODIPY with copper ions to form a BCu nanocore, which was then cloaked with natural killer (NK) cell membranes for tumor targeting (Figure 13A) (Yu et al., 2025). This platform enabled PDT under 658 nm light and mild PTT under 1064 nm irradiation (Figure 13B). The combined PDT/PTT not only killed tumor cells directly but also depleted GSH, which normally chelates copper, thereby amplifying cuproptosis. The synergy between phototherapy and cuproptosis triggers robust ICD, reprograms the immunosuppressive microenvironment, and enhances the body’s antitumor immune response.

**FIGURE 14:**
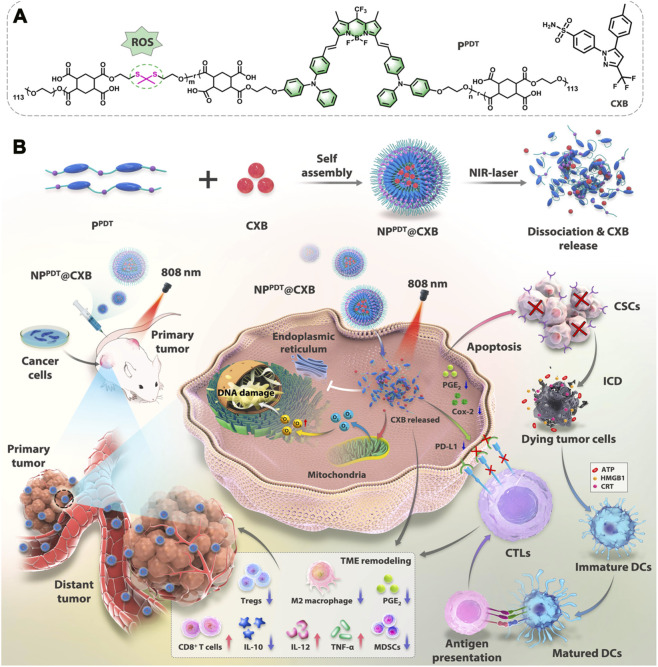
**(A)** Molecular structures of the BODIPY-based polymer and CXB. **(B)** Design strategy of the NPPDT@CXB nanoplatform and schematic illustration of drug release upon light irradiation and its combined antitumor effect. Reproduced with permission from Zhang X. et al., 2023. Copyright 2023 Elsevier Ltd.

Finally, beyond activating pro-inflammatory pathways, smart nanoplatforms can also counteract the negative feedback loops triggered by PDT itself (Chen et al., 2025). For instance, PDT-induced ICD is often accompanied by upregulation of the COX-2/PGE2 pathway and PD-L1 expression, creating an immunosuppressive “brake.” Shang *et al.* addressed this by synthesizing a biodegradable polymer, PPDT, containing BODIPY units and ketal linkages, and loading it with the COX-2 inhibitor celecoxib (CXB) to form NPPDT@CXB (Figure 14A) (Zhang X. et al., 2023). Their mechanistic studies confirmed that while PDT induced ICD, it simultaneously activated the COX-2/PGE2 pathway and increased PD-L1. In response, PPDT@CXB used light-generated ROS to cleave the ketal bonds, releasing CXB precisely at the tumor site. This suppressed PGE2 production and downregulated PD-L1 (Figure 14B). *In vivo*, PPDT@CXB under 808 nm irradiation showed potent inhibition of both osteosarcoma and ovarian cancer through combined phototherapy and immune activation. Critically, this strategy addresses a fundamental limitation of PDT, its tendency to induce immunosuppression. It delivers an important message for future photoimmunotherapy design: effective treatment must not only activate the immune system but also release the immune “brakes” imposed by the therapy itself. This synergy lies in BODIPY-mediated PDT activating ICD while CXB counteracts PDT-induced immunosuppression, ensuring sustained and robust antitumor immune responses.

## 5 Conclusion and perspectives

BODIPY-based photosensitizers have emerged as promising multifunctional platforms for cancer phototherapy and immunotherapy, with remarkable progress achieved in molecular design, intelligent delivery, and immune synergy. We have summarized the core advances of these systems, highlighted the critical bottlenecks hindering their clinical translation, and proposed future research directions and clinical outlook to facilitate their transformation into practical tools for precision cancer therapy.

### 5.1 Summary of BODIPY-based photosensitizers in cancer therapy

These BODIPY-based systems owe their tunable photophysical properties, high photosensitivity, and modifiable molecular structures to their role as ideal photosensitizers or carriers for integrating therapeutic and immune-modulating functions. Adopting molecular modification strategies such as cyclization, electronic cloud modulation, supramolecular activation, and metal coordination has effectively addressed the intrinsic limitations of conventional BODIPY dyes including low ROS generation, short absorption wavelength, and poor stability. At the delivery level, intelligent designs including O_2_ co-delivery, GSH depletion, hypoxia-responsive release, light-controlled drug uncaging, and membrane-targeting modification enable precise tumor accumulation and active remodeling of the tumor microenvironment to overcome the bottlenecks of poor targeting and insufficient local therapeutic concentration. In immunotherapy, synergistic approaches such as immune checkpoint blockade, pyroptosis or cuproptosis induction have successfully transformed localized photodynamic therapy into durable systemic immunity, breaking the limitations of single-modal therapy and achieving long-term tumor control. In summary, BODIPY-based photosensitizers through the precise integration of molecular design, intelligent delivery, and immune synergy construct efficient, multi-layered nanoplatforms for tumor treatment and demonstrate significant potential for clinical translation.

### 5.2 Challenges in clinical translation

Despite the notable advantages of BODIPY-based photosensitizers in enhancing PDT and photoimmunotherapy, their further development and clinical translation face several critical challenges. The contradiction between synthetic precision and batch-to-batch consistency is prominent because complex synthetic processes often lead to variability in photophysical properties and purity, which compromises experimental reproducibility and clinical reliability, while lacking standardized manufacturing protocols and comprehensive safety assessments also hinders regulatory approval. Scaling up these syntheses for industrial production further exacerbates these issues as minor changes in reaction conditions can result in significant differences in product performance, while the high cost of raw materials and complex purification steps limit economic feasibility. Balancing targeting efficiency and systemic toxicity requires optimization since although various targeting strategies improve tumor accumulation, some modifications may cause nonspecific adsorption, aggregation, or rapid clearance in systemic circulation to potentially induce toxicity to normal tissues. Moreover, the long-term impact of BODIPY-based nanocarriers on immune homeostasis remains unclear, which raises concerns about potential autoimmune side effects. Although adaptive delivery strategies are developed to address tumor heterogeneity, single-stimuli-responsive carriers often fail to adapt to diverse tumor types or spatial variations within a single tumor, while the depth limitation of optical windows restricts the applicability of BODIPY-based PDT to superficial tumors. Furthermore, the construction and regulation of multimodal synergistic therapies remain complex as integrating BODIPY with other therapeutic modalities always involves complicated synthesis steps, poor structural homogeneity, and insufficient synergy between different components. Finally, research on the impact on the immune microenvironment and long-term antitumor mechanisms is still insufficient because current studies focus primarily on direct tumor killing and short-term efficacy, while the detailed mechanism of immune synergy, long-term immune memory formation, and the mitigation of PDT-induced immunosuppressive feedback remain underexplored to limit their ability to combat tumor metastasis and recurrence.

### 5.3 Future research directions

To address these challenges, future research should advance from molecular design, delivery optimization, to clinical translation. Regarding synthetic precision and scalability, novel efficient synthesis strategies should be developed by leveraging computational chemistry and artificial intelligence to reduce synthetic variability. Exploring simple, low-cost synthetic routes and scalable purification techniques will improve batch-to-batch consistency and reduce production costs. For mitigating systemic toxicity and enhancing targeting specificity, multi-stage targeting strategies should be pursued to enhance tumor accumulation *via* passive and active targeting, followed by improved subcellular localization to maximize therapeutic efficacy and minimize off-target effects. To optimize long-term clearance and pharmacokinetics, modifying BODIPYs with hydrophilic, non-immunogenic polymers and designing biodegradable nanocarriers can extend blood circulation time, promote long-term clearance, and reduce chronic toxicity. For optimizing TME modulation and overcoming depth limitations, more efficient adaptive nanoplatforms need to be designed by integrating multiple triggers including hypoxia, pH, enzymes, redox status, and light to adapt to the dynamic TME, while exploring upconversion nanoparticles or X-ray-activatable photocages will extend the application of BODIPY-based PDT to deep-seated tumors. To simplify multifunctional integration and precise control, “all-in-one” BODIPY designs should be advanced by embedding photosensitizers, immune modulators, and targeting ligands directly into nanocarriers *via* one-pot synthesis to shorten synthetic routes and ensure uniform dispersion, while leveraging spatiotemporal differences of external stimuli to program the sequential activation of different therapeutic modes. Finally, research on immune synergistic therapy and long-term efficacy should be expanded by co-loading BODIPY nanocarriers with immune adjuvants and combining them with immune checkpoint inhibitors to enhance ICD-induced immune responses, activate dendritic cells and T cells to establish systemic antitumor immune memory, while utilizing the modifiability of BODIPYs to load reporter genes or contrast agents could enable real-time imaging monitoring of the treatment process to promote theranostics.

### 5.4 Clinical translation outlook

For the clinical translation of BODIPY-based photosensitizers, three key issues need to be solved including safety, scalability, and dosing. In terms of safety, a comprehensive preclinical evaluation system should be established with acute/chronic toxicity tests, long-term organ function monitoring, and immunogenicity evaluations, and corresponding mitigation strategies for potential toxicity and off-target effects must be verified in large animal models. Regarding scalability, current laboratory-scale synthesis methods are difficult to meet clinical demand so developing scalable synthesis technologies with low cost and good reproducibility is essential. In terms of dosing, personalized dosing methods considering patient characteristics, tumor type, and size should be developed, while clarifying the pharmacokinetic properties of BODIPY-based systems in different patients to avoid adverse reactions induced by individual differences. Additionally, as novel nanomedicines, BODIPY-based photosensitizers must comply with regulatory medical guidelines and criteria for safety and efficacy to accelerate the clinical translation process. In conclusion, with the integration of materials science, nanotechnology, and tumor biology, BODIPY-based photosensitizers are poised to evolve into intelligent, personalized, multimodal synergistic platforms for tumor therapy, ultimately achieving clinical translation.
